# Stable multiple sclerosis patients on anti-CD20 therapy should go on extended interval dosing—“Yes”

**DOI:** 10.1177/13524585211055593

**Published:** 2021-12-21

**Authors:** Leoni Rolfes, Sven G Meuth

**Affiliations:** Department of Neurology, Medical Faculty, Heinrich-Heine-University Düsseldorf, Düsseldorf, Germany; Department of Neurology, Medical Faculty, Heinrich-Heine-University Düsseldorf, Düsseldorf, Germany

## Introduction

Anti-CD20 antibodies such as rituximab proved powerful yet remained as an off-label treatment in multiple sclerosis (MS) until the approval of ocrelizumab in 2018. The drug-free intervals between two courses of B-cell depletion are long, as treatment efficacy is determined by prolonged immunosuppression, which can eventually be assessed by peripheral B-cell reconstitution. This provides the opportunity to individually delay therapy, known as extended interval dosing (EID), which is likely to be beneficial in view of safety concerns. Specifically, aside from frequent infusion reactions, continuous B-cell depletion might be associated with long-term immunological complications, such as an increased risk of malignancy or hypogammaglobulinemia. The latter, in turn, is likely to result in higher infection rates and reduced vaccine efficacy. Finally, EID may also provide sufficient time for a drug-free pregnancy, while under ongoing protection from disease activity.

In light of notable recent studies, a number of mechanistic features argue in favor of durable efficacy beyond the standard 6-monthly dosing; only ~20% of patients treated with rituximab and even fewer (~5%) with ocrelizumab began to repopulate by 6 months and CD19^+^ repopulation took longer than one year.^1^ Although the potential of anti-CD20 antibodies to deplete B-cell subsets has yet to be fully elucidated, both agents further deplete memory B-cells,^2^ which can last for years after treatment. Marked depletion of memory B-cells also appears to be a common feature contributing to the efficacy of so-called “immune reconstitution therapies” (IRT), which show long-term efficacy after short treatment cycles.

## Impact of EID of anti-CD20 therapies on disease activity and patient safety

Several studies showed that EID between two infusions of rituximab or ocrelizumab in MS is associated with a low risk of disease activity. A recently published large multicenter study compared ocrelizumab-treated MS patients on EID with a control group on standard interval dosing, 3 months after the last treatment cycle.^3^ There were no differences between both groups in terms of relapses, confirmed progression of disease or NEDA-3 status, suggesting that EID does not affect efficacy, at least after short-term evaluation. Similar findings were observed by Baker et al.^4^ for longer follow-up periods. Specifically, using data from the open-label, phase II ocrelizumab extension trial, they demonstrated that 12 to 18 months after the last infusion following three cycles of ocrelizumab, the levels of disease activity appear to be similar to that seen in the phase III extension studies following six cycles of ocrelizumab.^4^ Likewise, smaller real-world studies supported longer treatment-free intervals of ocrelizumab in MS without lack of efficacy.^5^ In line with this, the phase I extension study of rituximab in MS reported a maintained benefit 12 months after the last infusion.^2^ Moreover, off-label studies with rituximab, where treatment was halted, demonstrated a long-acting benefit and an absence of rebound disease activity after stopping therapy.^6,7^

Regarding safety concerns, one study showed that there were fewer overall adverse events and infections in the EID cohorts.^4^ In addition, it has recently been indicated that delaying anti-CD20 infusions by 3 to 6 months increases the likelihood of developing an adequate humoral response to COVID-19 vaccination.^8^

Taken together, these findings make a convincing argument that B-cell-depleting agents induce durable inhibition of relapsing disease. Consequently, it is likely that efficacy can be maintained by reducing the frequency of dosing, while limiting infections and other risks associated with continued immunosuppression and allowing for more effective vaccination.

## Personalized B-cell-based treatment regimens for time-to-infusion decision-making

It has been repeatedly hypothesized that continuous monitoring of peripheral CD19^+^ B-cells may be a sufficient guide for individualized decision-making regarding reinfusion intervals. Indeed, studies investigating rituximab in other autoimmune conditions have indicated that low levels of CD19^+^ B-cells serve as a surrogate marker to justify delaying B-cell-depleting infusions. However, evidence of personalized, B-cell-tailored EID in MS patients remains controversial: Although EID of anti-CD20 antibodies represented a predictor for repopulation of CD19^+^ B-cells in several studies,^5,7,9^ only one study showed that the therapeutic effect was closely associated with the absence of CD19^+^ B-cells.^9^ In contrast, no association between the absolute CD19^+^ B-cell number and re-emerging disease activity was evident in other studies,^3,7^ suggesting that the effects of rituximab and ocrelizumab in MS are maintained after CD19^+^ B-cell repopulation.

Of note, a more detailed assessment of B-cells in the periphery including CD19^+^ CD27^+^ memory B-cells was shown to be more reliable in the prediction of clinical activity. Indeed, a preliminary study with rituximab suggests that dosing according to memory B-cell population kinetics reduces dosing frequency while maintaining efficacy in MS.^10^ Monitoring of peripheral B memory cells further demonstrated biomarker activity in other autoimmune diseases, including neuromyelitis optica and myasthenia gravis.

## Conclusion

Overall, these findings indicate that EID can be performed without a detrimental impact on effectiveness, and while improving patient safety. Although the complex mechanisms underlying the observed effects remain unclear, the findings suggest that anti-CD20 antibodies likely possess features of an IRT.

A major challenge for MS research now is to elucidate the exact impact of anti-CD20 antibodies on the immune system to determine surrogate markers for individual time-to-infusion decision-making. In this context, the memory B-cell repopulation rate appears to be a promising candidate to appraise individually adapted therapy intervals.

Thus, we should further concentrate our research efforts on head-to-head studies of anti-CD20 therapy with extended doses compared to the current standard, including immunophenotyping analysis. Moreover, future prospective studies should investigate the long-term impact of continuous EID in terms of clinical outcomes and patient safety.

If the promising durable effect of CD20 depletion is confirmed, ocrelizumab would have a greater utility in the treatment of MS due to relatively low side effects and limited need for monitoring compared to other highly effective therapies. However, even if ocrelizumab requires repeated treatments, a lower dosing frequency is beneficial as it lessens the likelihood of infusion-related events and the development of severe infections.
